# Mycoremediation potential of *Pleurotus* species for heavy metals: a review

**DOI:** 10.1186/s40643-017-0162-8

**Published:** 2017-07-10

**Authors:** Meena Kapahi, Sarita Sachdeva

**Affiliations:** 1 Department of Biotechnology, Manav Rachna International University, Sector 43, Faridabad, 121004 India; 2 Manav Rachna University, Sector 43, Faridabad, 121004 India

**Keywords:** *Pleurotus* species, Heavy metals, Biosorption, Mycoremediation, Laccase, Manganese peroxidase

## Abstract

Mycoremediation is one of the biotechniques that recruits fungi to remove toxic pollutants from environment in an efficient and economical manner. Mushrooms, macro-fungi, are among the nature’s most important mycoremediators. *Pleurotus* species (also called oyster mushrooms) are considered to be the most popular and widely cultivated varieties worldwide and this might be attributed to their low production cost and higher yields. Apart from their nutritive and therapeutic properties, *Pleurotus* species have high biosorption potential due to their extensive biomass, i.e. mycelial production. The genus has been reported to accumulate high levels of heavy metals. The current state-of-the art review mainly summarises previous investigations carried out by researchers on different roles and mechanisms played by *Pleurotus* species on heavy metals mycoremediation.

## Introduction

Indiscriminate use of chemicals has led to severe contamination of environmental segments by heavy metals. Heavy metals are non-biodegradable and tend to be biomagnified in the food chain (Singh et al. 2008). They pose a risk to human health when transferred via food chain and can further lead to toxic effects in organisms even in trace amounts. These metals can hinder different cellular processes. Their effects are generally concentration dependent and also differ with respect to individual toxicity. Hence, it becomes crucial to remove them prior to final discharge of effluents in environmental segments.

Conventional methods like chemical precipitation, adsorption, ion exchange, reverse osmosis and electro-dialysis, to get rid of heavy metal burden of the environment, have their own shortcomings. These methods offer limitations like slow metal precipitation and incomplete removal (Aziz et al. 2015), generation of contaminated sludge requiring careful disposal (Gunatilake 2015; Ayangbenro 2017), high cost involved in the processes (Firdousi 2017), high energy and reagent requirements and clogging of membranes (Ahalya et al. 2003). In this scenario, it is important to opt for an economically feasible and effective treatment method which is free from these limitations and is able to translate the need of removal of heavy metals in terms of eco-friendly approach. Bioremediation is a way of cleaning up heavy metals using biomass (or microorganisms) through the processes of biodegradation, biosorption, bioaccumulation and bioconversion operating in different ways (Kulshreshtha et al. 2014; Mosa et al. 2016). The microorganisms bind to heavy metals and concentrate them (Joutey et al. 2015). Biosorption is a passive process and heavy metals get adsorbed on the surface of the biosorbent (Velásquez and Dussan 2009) exhibiting the tolerance of biosorbent towards heavy metals. The mechanisms like extracellular (chelation and cell wall binding) and intra-cellular (binding to compounds like proteins) sequestration of heavy metals have been proposed as mechanisms for heavy metals tolerance in fungi (Fawzy et al. 2017). Biosorbent from mushrooms can be prepared from mycelium or fruit body (live or dead) and spent mushroom substrate (SMS). The factors like the presence of microbial population, the availability of contaminants to these organisms, metal ion concentration and environmental factors like temperature, pH and the presence of nutrients affect the biosorption process in totality (Prakash 2017). The process includes precipitation, ion exchange, electrostatic interaction, the redox process, etc. (Yang et al. 2015).

The biological process of remediation display features like economic viability (Ayangbenro 2017) and repeated use of biomass, selective metal binding, effective desorption and recycling of desorbents. Different microorganisms like algae, bacteria, fungi, yeast have been employed to carry out biosorption. The potential of fungal biomass as biosorbent has been accepted for the removal of heavy metals and radionuclides from polluted waters because of their excellent metal binding properties and tolerance towards metals and adverse environment like diverse pH and temperature conditions (Qazilbash 2004; Anand et al. 2006; Yazdani et al. 2010; Salman et al. 2014). Fungi have been reported to exhibit the ability to chemically modify or affect their bioavailability (Prakash 2017). Fungi have chitin in their walls which can tolerate high concentrations of metals and are capable of growing on medium at low pH and temperature exhibiting excellent mycoremediation potential.

Mushrooms, macro-fungi, have fruiting bodies that grow out of a mass of mycelium. They are a favourite delicacy in many parts of the world. The consumption of edible mushrooms is increasing due to a good content of proteins and trace minerals. Mushrooms have also been reported as nutraceuticals having anti-oxidant, anti-cancer, immunostimulatory, anti-inflammatory and anti-diabetic therapeutic properties (Barros et al. 2007; Kim et al. 2007; Sarikurkcu et al. 2008; Synytsya et al. 2009). These functional characteristics are mainly due to their chemical composition.

Apart from this, mushrooms can be employed for decontamination of the polluted environment. Mushrooms can build up heavy metals in high concentrations in their bodies above maximum permissible concentrations (Kalac and Svoboda 2000) and can act as an effective biosorption tool (Das 2005). High accumulation potential and shorter life span are some of the advantages of using mushrooms as biosorbents. Mushrooms belonging to the genera including *Agaricus*, *Boletus*, *Armillaria*, *Polyporus*, *Russula*, *Pleurotus*, *Termitomyces* have been investigated by some researchers for the uptake of heavy metals (Raj et al. 2011).

## *Pleurotus* species

The genus *Pleurotus*, commonly called Oyster mushroom, is a type of gilled mushrooms which grows normally on wood. It encompasses many species, for example *P. ostreatus*, *P. pulmonary*, *P. sajor*-*caju*, *P. cornucopiae*, *P. sapidus*, *P. platypus* and *P. ostreatoroseus*. It is found all around the world, mainly in forest environments. The genus has enzymes like laccase (LAC) and Mn-peroxidase (MnP), which degrade the lignocellulosic residues into food and enable them to grow on a variety of agricultural wastes with broad adaptability to varied agro-climatic conditions (Agrahar-Murugkar and Subbuakshmi 2005). A number of substrates like wheat straw, corn and sawdust can be used for its cultivation. They are popular and are widely cultivated throughout the world for food owing to simple production technology and their higher biological efficiency (Manzi et al. 2001). The genus is considered to be rich in proteins, fibres, carbohydrates, vitamins and minerals and owns a very pleasant taste. It is rich in immense therapeutic properties (Kalac and Svoboda 2000). There has been a rise in research activities related to the genus because of its multiple uses including biosorption.

## *Pleurotus* species—sequestering heavy metals

*Pleurotus* species have been found to demonstrate a very effective biosorption potential for a wide range of environmental contaminants including heavy metals (Table 1). The accumulation of heavy metals in the fruit bodies tends to increase with an increase of the metals in the substrate (Ogbo and Okhuoya 2011). Heavy metals have become concentrated in certain areas, such as traffic congested highways, emission areas and cement- and battery-waste polluted sites. *Pleurotus* species growing near these polluted sites have the ability to accumulate heavy metals in high concentrations in their bodies. Mushrooms growing in heavily polluted areas like vicinity of the smelters have been reported to accumulate as much as 1540 times more than the background level of nickel (Barcan et al. 1998). The bioaccumulation potential of *P. ostreatus* from metal scrap sites has also been evaluated for Cu, Fe, Zn and Mn (Boamponsem et al. 2013). However, the accumulation potential of the species varies with the metallic species. Differences in accumulation potential for different heavy metals may be ascribed to the various types of growth substrates found in ecosystems. In a study conducted by Brunnert and Zadraz ˘il (1983), more Hg than Cd has been found to be accumulated in fruiting bodies in *P. ostreatus*, while more Cd has been found in *P. flabellatus*. Purkayastha et al. (1994) reported highest uptake of Cu and Cd as compared to Co and acid Hg ions by *P. sajor*-*caju*. However, the uptake of Cd was reduced in the presence of Cu in *P. sajor*-*caju* owing to the chemical interference.

## Biosorption from the substrates

They have the ability to enhance the nutritional content of the soil found in these areas (Adenipekun 2008) and bioremediate (Radulescu et al. 2010). A considerable decrease in Cu, Mn and Ni in cement-contaminated soil and a slow decrease in lead content of battery-polluted soils in case of *P. pulmonarius* have been observed (Adenipekun et al. 2011). The bioaccumulation potential of Hg by *P. ostreatus* grown on artificial compost has also been studied by Bressa et al. (1988). Uptake and bioaccumulation studies have been done on *Pleurotus* species grown on metal-enriched substrates (Jain et al. 1988, 1989). *Pleurotus sajor*-*caju,* grown on metal-enriched substrate duckweed, has been found to accumulate Cd content above permissible limits (Jain et al. 1988).

## Heavy metals distribution after biosorption

Subsequent to uptake, the metals are distributed unevenly within the fruiting bodies of mushrooms. The highest concentrations have been observed in the spore-forming part followed by rest of the cap and stipe (Gabriel et al. 1996). Cd has been found to be present in higher concentrations in caps (22–56 mg/kg dry wt) than in stipe (13–36 mg/kg dry wt) (Favero et al. 1990a) in *P. ostreatus.* The fruit body production has been found to be unaffected when exposed to a concentration up to 285 mg Cd/kg of dried substrate. Cadmium has been found to be accumulated to a higher concentration of 20 mg/g dry weight in *P. ostreatus* when grown in liquid cultures of malt broth (Favero et al. 1991). *Pleurotus* species have been found to show resistance to high Cd concentrations (Gabriel et al. 1996). Their capacity to accumulate the heavy metals can lead to their immobilisation but ingestion by other organisms can result in transfer along food chain (Osman and Bandyopadhyay 1999). The amounts of Pb, As, Fe, Cd and Hg in *P. ostreatus* available in the market (Accra, Ghana) have been found to be unsafe (Quarcoo and Adotey 2013).

## Factors affecting the biosorption process

It has generated interest in the researchers to use the species for biosorption of heavy metals from wastewater. Influence of a range of operational parameters like pH, temperature, biomass and initial metal ion concentrations and contact time have been considered while assessing their biosorption potential. The biosorption by the target species varies with the type of metal, its concentration and composition of substrate (Javaid and Bajwa 2008; Ogbo and Okhuoya 2011). In the biosorption study conducted by Adhikari et al. (2004), *P. florida* has been found to sorb heavy metals in the order of Cd > Cr and to accumulate 1.2–2.5% more Cd than *Fusarium oxysporum*, *Penicillium* species and *Aspergillus awamorii*. The dead biomass can bind metals at levels higher, equivalent to or lesser than live biomass depending on the method used to kill the biomass (Zhu et al. 2010). Boamponsem et al. (2013) reported that the age of the fungal fruiting body or its size is of less importance in the accumulation of heavy metals by mushrooms. The interval between the fructifications affects the same. *P. florida*, *P. ostreatus* and *P. djamour* recorded the highest maximum accumulation (1.63–2.58 ppm) in the third flush of fructification (Dulay et al. 2015).

*Pleurotus florida, P*. *ostreatus, P. sajor*-*caju, P. djamor, P. salmoneo*-*stramineus* have been reported to be affected by Pb. The concentration of 100 ppm resulted in the lowest mycelial growth (Dulay et al. 2015). *P*. *ostreatus* have carboxylic, amino, thiol, phosphate and hydroxide groups on the cell wall helping in the biosorption of heavy metals (Banerjee and Nayak 2007; Javaid et al. 2011). IR analysis of lyophilised cells *P. eryngii* revealed the presence of carboxylic, amino, hydroxyl and methyl groups (Joo et al. 2011). *P*. *ostreatus* and *P*. *sapidus* have been reported to show affinity towards Cu and Zn as compared to Cd and Pb (Ita et al. 2006, 2008). This is in consensus with reports by Zhu et al. (2010). However, fruiting bodies of *P. ostreatus* immobilised in calcium alginate were shown to be effective in removing Pb and Co from solution (Xiangliang et al. 2005, 2009). *P. ostreatus* displayed tremendous removal potential in the order of Ni > Cu > Cr > Zn ions from effluents of electroplating units (Javaid and Bajwa, 2008). *P. floridianus* and *P. sajor*-*caju* have been reported to exhibit affinity (biosorption efficiency) in the order of Cd > Zn > Ni > Pb > Cu > Fe (Lamrood and Ralegankar 2013). Uptake of heavy metals by *Azolla* species and its further translocation in *P. sajor*-*caju* have been studied by Jain et al. (1989). Javaid et al. (2011) conducted a study to assess the biosorption potential of *P. ostreatus* in single and multi-metal ion systems for Cr, Cu, Ni and Zn. Similarly, the SMS biosorbent of the species has been reported to exhibit higher selectivity for Ni than Cu in a bi-metal biosorption study conducted by Tay et al. (2016). *P. sajor*-*caju* has been demonstrated to remove metals like Cu, Fe, Mg, Mn, Zn (in the pro-degradant additive) on modified polyethylene films (Klein et al. 2012).

Both chemisorption and ion-exchange have been reported to be the involved mechanisms in metals biosorption. Lyophilised cells of *P. eryngii* showed higher bioconcentration values for Pb and Cd (Joo et al. 2011). Studies were conducted on removal of Pb, Zn, Cu and Mn from artificially contaminated soil using *P. tuber*-*regium.* More than 90% of the metals were removed. There was a significant increase in the level of heavy metals in the pileus of the mushroom after biosorption process (Oyetayo et al. 2012). It has been reported to show preference towards Fe, Al, Zn and Mn followed by Pb and Hg (Nnorom et al. 2013). It has further been reported that *P. tuber*-*regium* has more bioaccumulative properties when grown from spawn rather than from sclerotia (Oghenekaro et al. 2008). In the packed bed column study on Cd employing *P. platypus* using industrial wastewater, the effect of parameters like bed depth and flow rate has been assessed (Vimala et al. 2011a). Biosorption of Cd by *P. mutilus* in packed bed column has also been done by Khitous et al. (2015). The packed biosorbent can be used for three regeneration cycles. *Pleurotus* SMS has been employed in a fixed bed study to remove Mn(II) ions from aqueous solutions. Flow rate of 1 ml/min, bed height of 30 cm, and metal ion concentration of 10 mg/l have been found to be suitable for biosorption (Kamarudzaman et al. 2015). *Pleurotus* species have also been assessed for the removal of different heavy metals from chemical laboratory waste in the form of live mycelia (Arbanah et al. 2012, 2013). The highest biosorption efficiency for Fe and Cu has been found to be 80.52 and 45.20%, respectively (Arbanah et al. 2012). In a similar study conducted by Akyüz and Kirbað (2010), *P. eryngii* grown on various agro-wastes has been reported to show maximum uptake of K and the lowest of Cu contents.

The pH values of a solution should be considered as an important factor impacting the biosorption process. The pH influences the toxicity and solution chemistry of the heavy metals (Frutos et al. 2016), hydrolysis and complexation properties by bringing changes in ionic form (Deng et al. 2009). Hence, the ionic charge of the functional groups and the metal speciation at varied pH values affect biosorption process. Under acidic environment, positively charged metal ions get attached to the negatively charged biomass. Under high pH, metal ions precipitate as metal hydroxides (Hlihor et al. 2014). The optimum pH for live and heat-inactivated *P. sapidus e*ncapsulated in calcium alginate beads has been found to be 6 (Yalçinkaya et al. 2002). In a study assessing the potential of *P. ostreatus* as a biosorbent in removing Pb(II) from electroplating industrial wastewater, the maximum Pb(II) biosorption of 92% in aqueous solution has been achieved at an unadjusted pH of 5.2 (Tay et al. 2009). Similarly, pH range of 2.5–6 for the biosorption of Ni, Zn, Cr, Cu, Fe and Pb has been reported for *P. ostreatus* (Arbanah et al. 2012; Osman and Bandyopadhyay 1999; Tay et al. 2010). Tay et al. (2010) also carried out a study regarding the removal of Pb and Cu ions from aqueous solution. Cu(II) removal sharply increased from 38.21% at pH 2.0 to 81% at pH 5.0 in *P. cornucopiae* as reported by Danış (2010). The maximum biosorption of Pb(II) by *P. ferulae* with pH up to 3, temperature 30°, and initial metal concentration 100 mg/l has been reported by Adebayo (2013). Optimum biosorption of divalence cations [Ni(II) and Cu(II)] by *Pleurotus* mushroom SMS has also been reported to be between pH of 5 and 6 (Tay et al. 2012). Pre-concentration and determination of Cd(II) and Co(II) in vegetables, using *P. eryngii* immobilised on Amberlite XAD-16 as a solid-phase biosorbent, have also been reported by Özdemir et al. (2012). The optimum extraction conditions were determined at a pH of 6.0 for Cd(II) and 5.0 for Co(II). In a similar study, pH range of 4–5 has been optimised for *P. ostreatus* immobilised on Amberlite XAD-4 for the biosorption of Cr, Cd and Cu (Kocaoba and Arısoy 2011). In the research on hybrid of *P. sajor*-*caju* and sunflower waste biomass immobilised on sodium alginate, the maximum equilibrium uptake for lead was found to be at pH 4.5 (Majeed et al. 2012). *P. cornucopiae* has been used to remove Cr from aqueous solution with bubbling fluidised bed (Xu et al. 2016).

Pre-treating the biomass with heat, alkalies or acids has a significant effect on the biosorption process depending upon the type of metal and fungal species. Pre-treatment of living biomass by physical and chemical methods resulted in an improvement in cadmium biosorption in comparison with living biomass of *P. florida* (Das et al. 2007). Methods like freeze drying (FD), oven drying (OD) and sun drying (SD) have been used for *P. ostreatus* for analysing the contents of different heavy and trace elements. Among the detected elements, K ranked the highest by 2.59, 1.31 and 2.30% in FD, OD and SD samples, respectively. OD biomass of *P*. *ostreatus* showed an increase in removal rate on increasing metal ion concentration (Javaid and Bajwa 2007). The other conditions affecting the biosorption as reported are ionic strength, other ions and complexing agents. The presence of high ionic strength and appreciable quantities of a complexing agent like EDTA significantly reduce the Pb(II) removal (Osman and Bandyopadhyay 1999).

## Heavy metals vis-a-vis effects

The uptake of heavy metals has its consequent deleterious effects on the growth, productivity and cellular proteins. Gabriel et al. (1996) reported fructification of *Pleurotus* species in Cd-contaminated environment. Baldrian et al. (2000) demonstrated inhibition of mycelial penetration into soil by Cd and Hg. Effect of Hg on the highest cadmium uptake (between 88.9 and 91.8%) was observed with aerobic fungal biomass from the exponential growth phase in *P. sajor*-caju (Cihangir and Saglam 1999). Cadmium up to 150 µg/ml slowly inhibited mycelia development in case of *P. ostreatus* but never blocked it completely (Favero et al. 1991). Effect of Hg on the growth of wood-rotting basidiomycetes including *P. ostreatus* was studied by Mandal et al. (1998). The growth of the mushroom was significantly inhibited. Purkayastha et al. (1994) reported more than 85% reduction of growth in *P. sajor*-*caju* at 15 and 6 μg/ml of Pb(II) and Hg(II), respectively. Pb reduced mycelial protein significantly (36%), but Hg caused maximum reduction (30%) of proteins in sporocarps. Pb reduced biological efficiency of sporocarp production. Mercury has been reported to prevent growth and fruit body production in *P. tuber*-*regium*, while stipe length, stipe diameter and cap diameter were affected by lead followed by cadmium (Akpaja et al. 2012). Mineral (Fe, Zn, Li) enrichment reduced anti-oxidant activity in *P. ostreatus* owing to polyphenol complexation with these elements leading to decreased free radical availability (Fontes et al. 2013).

## Heavy metals and enzyme regulation

The saprotrophic basidiomycetes utilise a variety of extracellular enzymes including ligninolytic enzymes for the utilisation of complex nutrients (Kapoor and Viraraghavan 1998). Factors controlling enzyme production among white rot fungi have also been widely studied. The main factors that influence the enzyme production are the nutrients, inhibitory compounds, temperature and interrelationships with other fungi (Baldrian and Gabriel 2002). Extracellular ligninolytic and cellulolytic enzymes are regulated by heavy metals on transcription level and during the course of their action. The effect of the heavy metals on enzymatic activities influences the energy flux in the ecosystem. In a study, a positive regulation of laccase and isoenzymes on copper application has been reported in the case of *P. ostreatus* (Baldrian and Gabriel 2002; Palmieri et al. 2000). The Mn-peroxidase activity decreased with increasing Cd concentration, whereas activities of endo-1,4-l-glucanase, 1,4-l-glucosidase and laccase highly increased in the presence of metal (Baldrian and Gabriel 2003). It has been reported that the *P*. *sajor*-*caju* laccase isozyme genes (phenol oxidase A1b (POXA1b), POXA2 and POXC) are differentially regulated at the transcriptional level in response to copper and manganese (Collins and Dobson 1997; Soden and Dobson 2001). The addition of Hg has been found to decrease the activity of laccase immediately and reduce the stability of the enzyme (Collins and Dobson 1997; Baldrian and Gabriel 2002). Interestingly, Cu and also Hg increased MnP activity slightly. However, when incubated in the presence of all three metals, the activity of MnP decreased even at low concentrations of Cd, Cu and Hg (Baldrian and Gabriel 2002) showing the synergetic effect of the heavy metals. Manganese has also been found to affect MnP gene transcription and enzyme activity in a positive way in some fungi like *Pleurotus* spp. (Ruiz-Duenas et al. 1999). A study was conducted by Drzewiecka et al. (2010) to assess the effect morphology and physiology of *Pleurotus eryngii* after incubating the spawn in the Zn-, Cu-, Co-, Cd- and Ni-enriched substrate. Laccase activity was stimulated by Ni and Cu even at low concentrations during incubation stage; but inhibited during fruiting stage. The inhibition effect was more pronounced when exposed to multi-metal solution.

To consider a fungal species as a biosorbent, desorption of the adsorbed metal ions and subsequent reuse and efficiency of the biomass in biosorption need to be taken into account. Acidic solution desorption has been reported to be more effective than alkaline solution desorption (Prasad et al. 2013). Under acidic conditions, protons compete for the sites releasing metal ions in the medium. Ninety-seven percent desorption of the adsorbed Hg from immobilised and heat-treated *P. sajor*-*caju* resulted when eluted with HCl (Arica et al. 2003). Ninety-nine percent of lead could be desorbed from *P. ostreatus* using HCl for a contact period of 1 h. The used biomass of *P. florida* could be regenerated and reused for biosorption of lead for six times (Prasad et al. 2013). A regeneration rate of 59% of Cu has been reported for *P. mutilus* (Henini et al. 2011). However, they can be improved by coupling the chemical desorption method with a copper recovery; the regenerated biomass for a content 10 g/l has a maximum adsorption capacity smaller but still significant 59.75 mg/g.

## Conclusion

Different methods are being adopted to remove heavy metals from wastewater. Keeping in mind the financial aspects, it is necessary to produce low-cost, effective and recyclable adsorbents for their widespread use. There are some limitations of using mushrooms for biosorption. Biosorption potential of different species is also being assessed in a comparative way. Looking at the amount of work done on *Pleurotus* spp., the species holds a promise to be used as a biosorbent for heavy metals. The degree of tolerance is different for the species for different heavy metals. For performance assessment studies in the future, multi-component sorption studies should be stressed upon as the industrial wastewater is a cocktail of metal ions in solution and that plays an important role in the sorption efficiency of the species. The biosorption potential of the species is yet to be tapped and used commercially. Mushrooms being a food crop and looking at the potential of mushroom mycelia, the SMS produced after harvesting the mushroom can be used for the mycoremediation of the degraded sites. The aged mycelia, SMS, are otherwise generated in huge amounts by the mushroom farms and pose a disposal problem.

**Table 1:** Previous contributions of heavy metals biosorption using different forms of *Pleurotus* species

Biosorbent type	*Pleurotus* species	Heavy metals	References
Oven- and freeze-dried, autoclaved mycelia	*P. florida*	Cd	Das et al. (2007)
Oven-dried mycelia	*P. ostreatus*	Cr	Javaid and Bajwa (2007), Puentes-Cárdenas et al. (2012)
		Pb	Tay et al. (2009), Liew et al. (2010)
		Cd	Tay et al. (2011)
		Cu, Cr, Ni, Zn	Javaid et al. (2011)
	*P. florida*	Pb	Prasad et al. (2013)
Live mycelia	*P. ostreatus*	Cd	Favero et al. (1990a, b)
		Hg	Mandal et al. (1998)
		Cu, Cr, Ni, Zn	Javaid and Bajwa (2008)
		Cu, Cr, Fe, Zn	Arbanah et al. (2012)
		Cr	Arbanah et al. (2013)
	*P. ostreatus*, *P. florida*, *P. djamour*, *P. salmoneo*-*stramineus*, *P. cystidiosus*	Pb	Dulay et al. (2015)
	*P. eryngii*	Mn	Wu et al. (2016)
	*P. floridianus*, *P. sajor*-*caju*	Cu, Cd, Fe, Ni, Pb, Zn	Lamrood and Ralegankar (2013)
Biomass immobilised on calcium alginate	*P. ostreatus*	Pb	Xiangliang et al. (2005)
		Co	Xiangliang et al. (2009)
		Cu, Pb	de Almeida and Burgess (2013)
	*P. sapidus*	Cd, Hg	Yayçinkaya et al. (2002)
	*P. sajor*-*caju* and sunflower waste biomass hybrid	Pb	Majeed et al. (2012, 2014)
Biomass immobilised on XAD-4	*P. ostreatus*	Cu, Cr, Cd	Kocaoba and Arisoy (2011)
	*P. eryngii*	Cd, Co	Özdemir et al. (2012)
SMS	*P. ostreatus*	Cu	Tay et al. (2010)
		Cr	Carol et al. (2012)
		Cu, Ni	Tay et al. (2012, 2016)
		Cd, Pb, Cu	Frutos (2016)
Fruit body accumulation	*P. ostreatus*	Cd	Favero et al. (1990a, b)
		Hg	Bressa et al. (1988)
	*P. cornucopiae*	Cu	Danis (2010)
		Cr	Xu et al. (2016)
	*P. platypus*	Cd	Vimala and Das (2011b)
	*P. ostreatus*, *P. tuber*-*regium.*	Hg	Nnorom et al. (2012)
	*P. ferulae*	Pb	Adebayo (2013)
	*P. ostreatus*, *P. florida*, *P. djamour*, *P. salmoneo*-*stramineus*, *P. cystidiosus*	Pb	Dulay et al. (2015)
	*P. eryngii*	Pb	Jiang et al. (2016)
	*P. ostreatus*	Pb	Jiang et al. (2017)
Sun-dried fruit	*P. ostreatus*	Pb	Osman and Bandyopadhyay (1999)
Oven-dried fruit	*P. ostreatus*	Cu	Huo et al. (2011)
		Cu, Pb, Zn, Mn	Oyetayo et al. (2012)
	*P. platypus*	Cd	Vimala and Das (2011b)
	*P. eous*	Cr, Ni, Pb	Suseem and Mary Saral (2014)
Freeze-dried fruit	*P. eryngii*	Cd, Pb	Joo et al. (2011)
